# Factors influencing the COVID-19 pandemic situation in Indonesia, Malaysia and Taiwan in 2021

**DOI:** 10.1016/j.puhip.2022.100311

**Published:** 2022-09-02

**Authors:** Rahayu Lubis, Fauzi Budi Satria, Rafdzah Ahmad Zaki, Nurjazuli Nurjazuli, Lucia Yovita Hendrati

**Affiliations:** a Faculty of Public Health, Universitas Sumatera Utara, Indonesia; b Ph.D. Program in Global Health and Health Security, College of Public Health, Taipei Medical University, Taiwan; c Centre for Epidemiology and Evidence-Based Practice, Faculty of Medicine, University of Malaya, Malaysia; d Faculty of Public Health, Diponegoro University, Indonesia; e Faculty of Public Health, Airlangga University, Indonesia

**Keywords:** COVID-19, Global pandemic, Vaccine effectiveness, Health security, Global health

## Abstract

**Objectives:**

The COVID-19 pandemic started over 2 years ago and spread rapidly throughout the world. The total number of cases and deaths is still increasing and the situation remains active across the globe. In the Asian region, COVID-19 vaccination began in early 2021; however, the COVID-19 situation remains uncertain. This study aims to compare the factors that influenced the COVID-19 pandemic situation in three countries in Asia (namely, Indonesia, Malaysia and Taiwan) throughout 2021.

**Study design:**

This ecological study utilises the data from the ‘Our World in Data’ website.

**Methods:**

In this study, the COVID-19 pandemic situation in each country is described by looking at the average daily number of deaths and cases per million population throughout 2021. A paired *t*-test was conducted to compare the significance of differences in the pandemic situation between 2020 and 2021. In addition, the COVID-19 vaccination profiles throughout 2021 were investigated. A multiple linear regression analysis was then performed to develop models to explain the factors influencing the COVID-19 pandemic situation in these three countries.

**Results:**

The COVID-19 pandemic situation in Indonesia, Malaysia and Taiwan in 2021 is significantly different from 2020. Malaysia had the highest COVID-19 vaccination coverage (79.4%), followed by Taiwan (78.5%) and Indonesia (58.3%). This study found that the following three factors consistently influenced the number of deaths and cases in these three countries [1]: positivity rate [2]; number of tests per 1000 population; and [3] number of tests per case.

**Conclusions:**

Although the severity of the COVID-19 pandemic situations in Indonesia, Malaysia and Taiwan was different, it is significantly influenced by the quality and quantity of COVID-19 testing and screening, in addition to the vaccination programmes and restriction policies implemented in each country. As a result of the ability of the SARS-CoV-2 virus to mutate, it is recommended that each country strengthen their comprehensive approach to have an effective and efficient coping strategy for the COVID-19 pandemic.

## 1 Introduction

The COVID-19 pandemic shocked the world when the incidence of confirmed cases and deaths rapidly increased beyond expectations. At the end of 2021, COVID-19 had infected over 300 million people and caused more than 5 million confirmed deaths worldwide [1]. As of 30 June 2022, there were 544,324,069 confirmed cases of COVID-19, including 6,332,963 deaths reported to the World Health Organisation (WHO). Many prevention strategies have been implemented throughout the world to control the COVID-19 pandemic, including COVID-19 vaccines [2,3]. The COVID-19 vaccine is considered to be the safest way to bring this pandemic to an end since all of the vaccinated population will have some immunity to the virus. Within 12 months of the beginning of the COVID-19 pandemic, several types of COVID-19 vaccines had been approved for use in humans [4]. In January 2021, Russia (Sputnik vaccine) and China (Sinovac vaccine) launched their COVID-19 vaccine programmes. Four COVID-19 vaccines were authorised by the European Medicines Agency in April 2021: Spikevax (mRNA-1273, Moderna by Cambridge, US) and Comirnaty (BNT162b2, BioNTech-Pfizer, Mainz, Germany/New York, United States) are based on mRNA, while Vaxzevria (ChAdOx1 nCoV-19, Oxford-AstraZeneca, Cambridge, United Kingdom) and Janssen vaccine (Ad26.COV2–S, Janssen-Cilag International NV, Beerse, Belgium) are adenovirus-based vector vaccines. Two doses of the vaccines are needed for full vaccination, with the exception of the Janssen vaccine that only requires one dose [5,6]. Globally, commencement of the initial vaccination period varied by country, as did the type of vaccine used; however, among Asian countries, COVID-19 vaccination started almost simultaneously. In Indonesia, the COVID-19 vaccination programme started in January 2021, while in Malaysia it was February 2021 and in Taiwan March 2021. However, the average daily number of vaccinations varied between these three countries. The coverage of at least one dose of COVID-19 vaccination between March and October 2021 in Indonesia reached 50%, while it is 40% and 70% in Malaysia and Taiwan, respectively [4].

Currently, the most challenging aspect of pandemic control is the mutation of the SARS-CoV-2 virus. At least five variants of COVID-19 have been identified globally. The Alpha and Beta variants were identified at the end of 2020, while the Gama-variant was discovered in early 2021. Then, in the middle of 2021, the Delta variant spread globally and resulted in an increase of COVID-19 cases and deaths worldwide. At the end of 2021, when the Delta variant has been declining, another virus mutation known as the Omicron variant started spreading globally [7]. In some regions, COVID-19 cases are starting to increase again and the need for booster vaccination is highly recommended [8,9]. This situation has raised the issue of equality as many countries, especially low- and middle-income countries, are still struggling to secure the first dose of the COVID-19 vaccine [10]. This study aims to compare the factors that influence the COVID-19 pandemic situation in three countries in Asia (namely, Indonesia, Malaysia and Taiwan) throughout 2021.

## 2 Methods

### 2.1 Study design and setting

This is an ecological study utilising data from the ‘Our World in Data’ website [11]. To measure the COVID-19 situation, the daily number of cases per million (NC) and the daily number of deaths per million (ND) were selected as the dependent variables. We used two dependent variables (NC and ND) to describe the COVID-19 situation based on its morbidity and mortality. The study adjusted for the sizes of the populations, as the differences in the population size between Indonesia, Malaysia and Taiwan are quite significant [12,13].

The following six factors were proposed as the independent variables in this study that might influence the COVID-19 situation [1]: the daily number of vaccination (NDV) [2]; case fatality rate (CFR) [3]; stringency index (SI) [4]; positivity rate (PR) [5]; the daily number of tests per 1000 population (TPT); and [6] the daily number of tests per case (TPC).

In this study, the NDV represents the number of people who had received at least one dose of the COVID-19 vaccine (not the number of people who are fully vaccinated against COVID-19). For countries that did not provide the daily number of people who received the COVID-19 vaccination, a manual calculation was performed by subtracting the total number of people vaccinated on that day from the total on the previous day [14].

The CFR is the number of confirmed deaths divided by the number of confirmed cases. The CFR indirectly reflects the performance of the healthcare system. However, as the number of actual cases and deaths is not accurately known and because countries that do very little testing may have fewer cases than the true number of confirmed cases indicated in the report, the CFR will overestimate the risk of death and it is not reflective of the real situation [15]. However, the CFR can theoretically be used to estimate the true number of infections. For example, if the CFR is triple the expected number, this might point to triple the reported number of infections. Typically the real CFR would lag some weeks behind the daily incidence and cannot be compared with the daily incidence as death takes some time to occur [16].

Definition of a COVID-19 death varies between countries. This study used PR, TPT and TPC to describe how COVID-19 testing was carried out by each country. The PR is the proportion of positive test results from the total number of tests taken. PR is the key variable to measure how adequately countries are testing and to gain understanding of the spread of the virus. In countries with a high PR, the number of confirmed cases is likely to represent only a small fraction of the true number of infections. PR can also suggest the virus is spreading faster than the growth seen in confirmed cases. The epidemic is under control when the PR is less than 5% in a country [17]. Furthermore, the TPT is the daily number of COVID-19 tests per 1000 population. This variable was used instead of the real number of tests as Indonesia has a very large population size. In addition, the TPC in the current study also describes the number of daily tests required to find a confirmed case of COVID-19 in a country [17].

The SI refers to the restriction policies to control the pandemic in each country. The SI is a composite measure based on nine response indicators including school closures, workplace closures, cancellation of public events, restrictions on public gatherings, closures of public transport, stay-at-home requirements, public information campaigns, restrictions on internal movements and international travel controls. The SI variable is used to record how strict the government policies are within each country. The SI does not measure or imply the appropriateness or effectiveness of a country's response. A higher SI score does not necessarily mean that a country's response is ‘better’ than others with a lower score on the index [18].

### 2.2 Data analyses

**Fig. 1:**
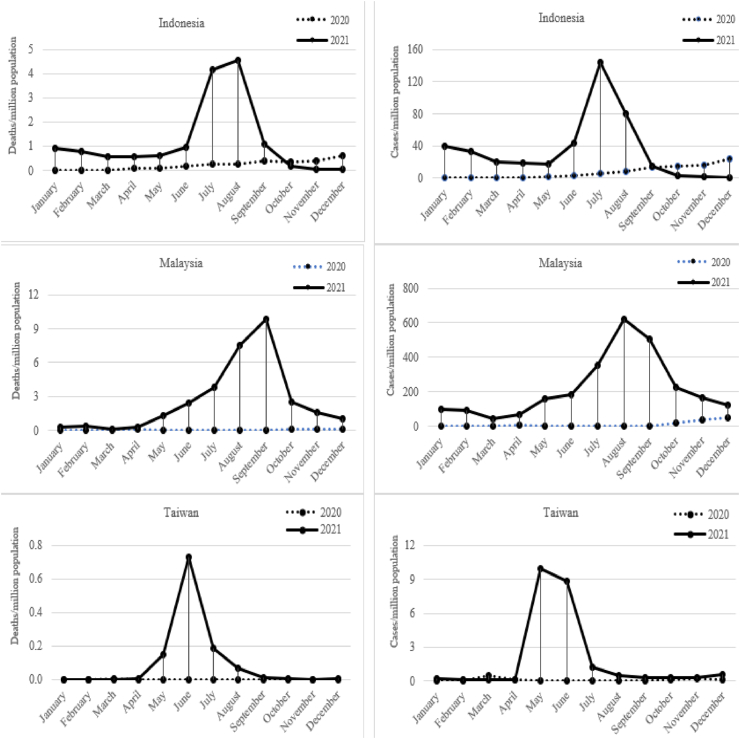
The average daily number of COVID-19 deaths and cases (i.e. trend of COVID-19 situation) in Indonesia, Malaysia and Taiwan in 2020 and 2021.

**Fig. 2:**
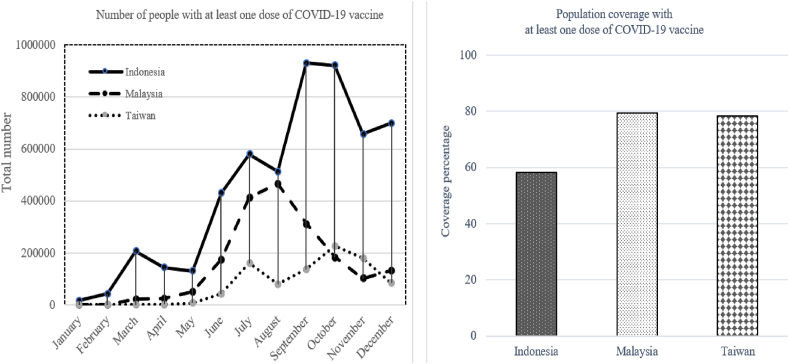
COVID-19 vaccination profile for Indonesia, Malaysia and Taiwan in 2021 (at least one dose).

Table 1 shows the comparison of the COVID-19 situations in Indonesia, Malaysia and Taiwan in 2020 and 2021. A paired *t*-test was then to determine whether the mean value of ND and CD for the two years differed significantly in each country. Fig. 1 illustrates the trend of the average daily number of cases and deaths in Indonesia, Malaysia and Taiwan in 2021. Fig. 2 shows the trend and coverage of vaccination among the three countries.

**Table 1:** Comparison of the COVID-19 pandemic situation in Indonesia, Malaysia and Taiwan in 2020 and 2021.

	Mean		Median		Std. Deviation		Minimum		Maximum		*t-score*
	2020	2021	2020	2021	2020	2021	2020	2021	2020	2021	
***Indonesia***											
*ND*	0.22	1.21	0.19	0.64	0.19	1.62	0	0	0.93	7.49	−11.7 *
*NC*	7.37	34.89	4.71	20.69	7.68	42.3	0	0	30.3	205.4	−11.6 *
***Malaysia***											
*ND*	0.04	2.59	0.00	1.43	0.06	3.15	0.00	0.03	0.34	18.06	−15.7 *
*NC*	9.45	221.1	1.16	159.2	16.1	183.5	0.00	28.7	77.04	750.5	−22.9 *
***Taiwan***											
*ND*	0.0008	0.09	0.00	0.00	0.008	0.25	0.00	0.00	0.13	1.55	−7.5 *
*NC*	0.092	1.86	0.042	0.29	0.18	4.84	−0.08	0.00	1.13	30.31	−7.4 *

NC, daily number of cases per million; ND, daily number of deaths per million.

*p < 0.01.

A multiple linear regression analysis was performed to identify the factors that influenced the COVID-19 situation in Indonesia, Malaysia and Taiwan in 2021. As there were two dependent variables, two models were developed for each country. The first model describes the factors that influenced the number of deaths, while the second model shows the factors that influenced the number of cases. The results of the first and second model for each country can be seen in Table 2 and Table 3, respectively.

**Table 2:** Results of multiple linear regression analysis for the daily number of deaths per million (ND) in Indonesia, Malaysia and Taiwan in 2021.

Country							
INDONESIA	R	R2		Adjusted R2		F (sig)	
	0.918	0.843		0.84		384.3*	
Coefficient	B	Std. Error	Beta	t (sig)	Correlation	Tolerance	VIF
**(Constant)**	−2.69	0.12	–	−22.13*	–	–	–
*PR*	18.09	0.49	0.93	37.02*	0.79	0.69	1.44*
*TPT*	3.29	0.24	0.41	13.78*	−0.05	0.51	1.98*
*CFR*	24.94	1.86	0.29	13.4*	0.22	0.88	1.14*
*NDV*	−3.17	0.00	−0.078	−2.98*	0.02	0.65	1.55*
*TPC*	0.00	0.00	−0.076	−2.71*	−0.34	0.56	1.79*
MALAYSIA	R	R2		Adjusted R2		F (sig)	
	0.97	0.95		0.95		1342.9*	
Coefficient	B	Std. Error	Beta	t (sig)	Correlation	Tolerance	VIF
**(Constant)**	−8.79	0.351	–	−25.07*	–	–	–
*PR*	99.02	2.81	1.11	35.23*	0.92	0.14	6.96*
*TPT*	0.23	0.05	0.08	4.32*	0.63	0.39	2.54*
*CFR*	224.7	9.98	0.39	22.49*	0.81	0.47	2.12*
*NDV*	−2.85	0.00	−0.15	−5.95*	0.75	0.23	4.31*
*TPC*	0.14	0.01	0.43	16.94*	−0.74	0.22	4.65*
TAIWAN	R	R2		Adjusted R2		F (sig)	
	0.74	0.55		0.54		86.7*	
Coefficient	B	Std. Error	Beta	t (sig)	Correlation	Tolerance	VIF
**(Constant)**	−0.1	0.025	–	−4.04*	–	–	–
*SI*	0.002	0.001	0.185	3.14*	0.573	0.36	2.76
*PR*	12.26	2.18	0.261	5.62*	0.501	0.58	1.71
*TPT*	0.27	0.037	0.497	7.35*	0.434	0.28	3.63
*TPC*	−6.12	0.00	−0.304	−5.83*	−0.281	0.47	2.15
*NDV*	−2.3	0.00	−0.112	−2.41**	−0.076	0.59	1.7

CFR, case fatality rate; NDV, daily number of vaccination; PR, positivity rate; SI, stringency index; TPC, daily number of tests per; TPT, daily number of tests per 1000 population.

*p < 0.01, **P < 0.05.

**Table 3:** The results of multiple linear regression analysis for describing daily number of cases per million (NC) in Indonesia, Malaysia, and Taiwan in 2021.

Country							
INDONESIA	R	R2		Adjusted R2		F (sig)	
	0.97	0.93		0.93		1249.2*	
Coefficient	B	Std. Error	Beta	t (sig)	Correlation	Tolerance	VIF
**(Constant)**	−49.09	2.06	–	−23.84*	–	–	–
*PR*	503.6	8.34	0.99	60.38*	0.881	0.69	1.44*
*TPT*	100.12	3.61	0.47	27.73*	0.031	0.64	1.56*
*TPC*	−0.02	0.003	−0.102	−5.59*	−0.378	0.56	1.79*
*CFR*	−151.6	31.11	−0.07	−4.87*	−0.123	0.92	1.09*
MALAYSIA	R	R2		Adjusted R2		F (sig)	
	0.982	0.965		0.965		1658.6*	
Coefficient	B	Std. Error	Beta	t (sig)	Correlation	Tolerance	VIF
**(Constant)**	−180.1	29.7	–	−6.07*	–	–	–
*PR*	5006.5	135.7	0.96	36.9*	0.96	0.14	6.97*
*TPT*	29.49	2.83	0.18	10.4*	0.71	0.32	3.13*
*CFR*	−5013.4	508.34	−0.15	−9.86*	0.61	0.42	2.36*
*TPC*	2.63	0.44	0.14	6.02*	−0.81	0.18	5.63*
*NDV*	0.00	0.00	0.15	7.02*	0.87	0.22	4.64*
TAIWAN	R	R2		Adjusted R2		F (sig)	
	0.933	0.871		0.869		1050.6*	
Coefficient	B	Std. Error	Beta	t (sig)	Correlation	Tolerance	VIF
**(Constant)**	−4.09	0.26	–	−16.02*	–	–	–
*PR*	831.56	22.53	0.91	36.91*	0.862	0.59	1.68*
*TPT*	2.46	0.36	0.23	6.92*	0.316	0.32	3.14*
*TPC*	0.00	0.00	0.11	3.98*	−0.295	0.49	2.06*
*SI*	0.02	0.007	0.09	3.01*	0.503	0.37	2.69*

CFR, case fatality rate; NDV, daily number of vaccination; PR, positivity rate; SI, stringency index; TPC, daily number of tests per case; TPT, daily number of tests per 1000 population.

*p < 0.01.

## 3 Results

### 3.1 Describing the COVID-19 situation in Indonesia, Malaysia and Taiwan

From Table 1, it can be seen that ND and NC in Indonesia, Malaysia and Taiwan in 2021 increased significantly (p < 0.001) compared to 2020. Among the three countries, Malaysia had the highest average daily ND (2.59) and NC (221.09), followed by Indonesia (ND 1.21; NC 34.89) and Taiwan (ND 0.09; NC 1.86).

Fig. 1 shows that the average daily number of COVID-19 deaths and cases started to increase in May 2021 in the three countries. In Taiwan, the average daily COVID-19 cases peaked in May 2021 and slowly declined in June, then decreased rapidly in July and continued to decline in the following months. Meanwhile, in Indonesia, the peak daily average number of COVID-19 cases occurred in July 2021 and continued to decline from August 2021 until the NC in October 2021 was lower than the NC in the previous year. In Malaysia, the average daily COVID-19 cases were at the highest in August 2021 and continuously decreased from September 2021. The average daily number of COVID-19 deaths was found to be highest in June, August and September 2021 in Taiwan, Indonesia and Malaysia, respectively. In Taiwan, the ND in September 2021 was similar to the previous year, while for Indonesia and Taiwan this was the situation in October and December, respectively.

### 3.2 Vaccination profile in Indonesia, Malaysia and Taiwan

Fig. 2 shows that 79.4% of the population in Malaysia received at least one dose of the COVID-19 vaccination by the end of 2021; this was 78.5% and 58.3% in Taiwan and Indonesia, respectively. The highest average NDV was different in the three countries. In Indonesia, the highest NDV was in September and October 2021, while in Malaysia NDV was highest in July and August, and in Taiwan it was in July and October 2021.

### 3.3 Factors influencing ND and NC in Indonesia, Malaysia and Taiwan in 2021

Table 2, Table 3 show results of the multiple regression analyses. Table 2 describes the factors that influenced the ND in 2021, while Table 3 described the factors that impacted NC in 2021. From Table 2, it was found that a combination of PR, TPT, CFR, NDV and TPC might explain 91.8% and 97% ND in Indonesia and Malaysia, respectively. Meanwhile, 74% of ND in Taiwan could be influenced by SI, PR, TPT, TPC and NDV.

Table 3 shows that the factors that influenced the NC in 2021 varied by country. In Indonesia, 97% of NC was described by PR, TPT, TPC and CFR. Meanwhile, in Malaysia NC was influenced by PR, TPT, CFR, TPC, NDV and SI. While in Taiwan, NC was affected by PR, TPT, TPC and SI.

## 4 Discussion

This study found that the average daily number of COVID-19 deaths and cases in Indonesia, Malaysia and Taiwan significantly increased in 2021 compared with 2020. The majority of the increase in COVID-19 deaths and cases was seen between May and September 2021. This dramatic increase was possibly due to the Delta variant of SARS-CoV-2 that began to spread during that period in the three countries. The Delta variant was first identified in India in October 2020 and the WHO subsequently named it as a variant of concern (VOC) in May 2021 [7]. Although SARS-CoV-2 has mutated several times since early 2020, the Delta variant has reportedly caused more infections and spreads faster than any other variant [19]. It is believed that the Delta variant reached Indonesia in March 2021 and by June 2021 this variant was responsible for 90% of COVID-19 [20]. Meanwhile, in Malaysia, the highest average daily number of COVID-19 cases occurred in July 2021. During this period, COVID-19 cases surpassed 1 million cases for the first time. The Delta variant was partly responsible for the surge as other countries in the Southeast Asia region also experienced similar increases [21].

In contrast with Indonesia and Malaysia, the COVID-19 cases and deaths in Taiwan were extremely low in 2020. This happened as Taiwan closed its borders very early and implemented a 14-day quarantine for anyone who arrived from abroad. However, after having a COVID-free period for >1 year, in May 2021 community outbreaks occurred and caused a spike of COVID-19 cases and deaths in Taiwan. This sudden outbreak was believed to be mostly influenced by the low coverage of COVID-19 vaccination and the easing of border control policies. Only around 1% of the population had been vaccinated against the virus in at this time. In addition, the Taiwan Government allowed the crews of Taiwanese airlines to quarantine at home for just 3 days. A week later, one of its crew tested positive in Australia followed by clusters of infections among the workers. Then, in May 2021, another pilot who previously visited a pub and restaurant in Taipei tested positive in the US. Since then, all mitigation strategies were considered too late as the number of cases and deaths continued to increase [22]. However, although the COVID-19 burden in Taiwan was relatively lower than in Indonesia and Taiwan, based on Fig. 1, the worst COVID-19 situation in Taiwan also happened around May and June 2021, the same period when the Delta variant was firstly reported in Taiwan [23].

Regarding vaccination among the three countries, the initiation of the vaccination programmes was different. In Indonesia, the initiation period was January 2021, while it was February and March 2021 in Malaysia and Taiwan, respectively [14]. Although Indonesia began earlier, it had the lowest coverage (53.8%). Meanwhile, Malaysia had the highest proportion of the population vaccinated with at least one dose of vaccination (79.4%), followed by Taiwan whose coverage was only slightly lower (78.5%). From Fig. 2, it can be seen that the vaccination rate increased significantly from May 2021 in all three countries. Furthermore, the current study found that the NDV significantly influenced the death burden in Indonesia, Malaysia and Taiwan (β = −0.08, β = −0.15, β = −0.11, respectively; p < 0.01), but it NDV only influenced the case burden in Malaysia (β = 0.15, p < 0.01). These findings are consistent with other studies that showed that one dose of vaccination was 77% effective against Delta-variant infection. Moreover, a full dose of vaccination was shown to be 97.5% effective against hospital admission due to Delta-variant infection [24]. There is a lag period between vaccinations and deaths as the immune system needs to be boosted sufficiently to prevent death [25]. Based on these results, it can be seen why the number of vaccinations influenced the number of deaths more than the number of cases.

From the models developed in this study, it can be seen that the SI did not significantly influence the number of deaths in Indonesia and Malaysia, but SI was the most important factor in Taiwan as it had the highest correlation (0.57, p < 0.01) compared with other factors. In addition, SI also significantly influenced the number of cases in Malaysia and Taiwan, but not in Indonesia; thus, restriction policies, such as border control, closure of public areas, schools and offices, had an impact in Malaysia and Taiwan. The current results are in line with other studies showing lockdowns to have a small but significant effect (β = 0.112, p < 0.01) in reducing the number of cases per million. In addition, one study showed that the SI produced the most important effect for mortality and infection rates (β = 0.588, β = 0.702, β = 0.518, β = 0.681; p < 0.01) [26].

The current study results found that the number of deaths and cases was consistently influenced by how COVID-19 testing was carried out in each country [17]. From Table 2, Table 3, it can be seen that three factors consistently influenced the number of deaths and cases in Indonesia, Malaysia and Taiwan; namely, PR, TPC and TPT. PR consistently had the highest correlation with the number of deaths and cases in the three countries among these three factors. Consistent with the current results, a previous study also stated that PR had a significant relationship with the numbers of deaths and intensive care unit (ICU) patients. In addition, to measure the spread of COVID-19, PR was a better indicator than the number of confirmed cases [27].

### 4.1 Limitations

Individual daily data were used to compute means in the analysis; however, the nature of infectious diseases means that daily counts might not be completely independent. The dependent variable is also influenced by other factors outside the variables studied. The secondary dataset contains raw data over time and it could be possible that some of the independent variables lag with the outcome.

## 5 Conclusions

Although the severity of the COVID-19 pandemic situation in Indonesia, Malaysia and Taiwan throughout 2021 was different, it is significantly influenced by the quality and quantity of COVID-19 testing and screening, as well as the vaccination programmes and restriction policies implemented in each country. As a result of the ability of the SARS-CoV-2 virus to mutate, it is recommended that each country strengthen their comprehensive approach to have an effective and efficient coping strategy for the COVID-19 pandemic.

## Ethical approval

Ethical approval was obtained from an institutional review board (IRB) from the Universitas Sumatera Utara with reference number 1094/KEP/USU/2021.

## Funding

This research supported by the World Class University (WCU)-Cluster knowledge project of the BPPTNBH 10.13039/501100013375Universitas Sumatera Utara No. 14594/UN5.1.R/PPM/2021.

## Declaration of competing interest

The authors declare that they have no known competing financial interests or personal relationships that could have appeared to influence the work reported in this paper.
